# Evaluating Slit-Lamp Performance in Diagnosing Anterior Uveitis: A Simulated Model Approach – Are All Slit-Lamps the Same, and Does it Matter?

**DOI:** 10.22599/bioj.577

**Published:** 2026-11-04

**Authors:** Kwok Kit Johnny Ma, Martin Rhodes, Joe Baxter, Thomas Evans, Jessy Choi

**Affiliations:** 1 University of Sheffield, UK; 2 Sheffield Teaching Hospitals NHS Foundation Trust: Sheffield, South Yorkshire, UK; 3 Sheffield Children’s NHS Foundation Trust, UK

**Keywords:** slit lamp, uveitis, anterior uveitis

## Abstract

**Background/Objectives:**

Anterior uveitis is diagnosed by detecting inflammatory cells in the anterior chamber using slit lamp biomicroscopy. Available slit lamps can vary significantly in illumination quality, potentially affecting diagnostic accuracy. This study evaluates device-to-device variability in slit lamp illumination within a tertiary ophthalmic centre and assesses its impact on anterior uveitis detection.

**Subjects/Methods:**

43 Haag-Streit BQ900 slit lamps (21 tungsten, 22 LED) were evaluated. Lux emissions were quantified using a calibrated photometer under standardised conditions. Simulated anterior chambers were developed using particles (~15 μm diameter) suspended in ethanol, targeting cell concentrations according to Standardisation of Uveitis Nomenclature (SUN) criteria grades 0–4+. Thirteen masked observers graded each chamber under standardised settings, comparing the dimmest tungsten and LED lamps. Statistical analyses included coefficient of variation (CV), independent and paired t-tests and Bland-Altman.

**Results:**

All slit lamps demonstrated variability in lux output, even among identical models. LED slit lamps exhibited a markedly higher maximum brightness (some >200,000 lux) than tungsten models (max 126,400 lux). Under diagnostic conditions (1 × 1 mm beam, 45°), LED slit lamps emitted significantly higher and more consistent lux compared to tungsten lamps (LED mean: 1275 ± 503 lux; tungsten mean: 793 ± 438 lux; p = 0.0017). In anterior chamber simulations, LED illumination improved detection at lower SUN grades (0.5+: LED mean = 2.23 cells, tungsten = 0.77 cells, p = 0.00006; 1+: LED = 5.77, tungsten = 4.15, p = 0.0403).

**Conclusions:**

Slit lamp illumination varies substantially. In a simulated model, lower illuminance was associated with reduced detection of low-grade inflammation. Routine illumination auditing and equipment standardisation, considering the broader adoption of LED slit lamps, may improve the reliability of uveitis assessment.

## Introduction

Uveitis is inflammation of the uveal tract, comprised of the iris, ciliary body, and choroid. It remains a significant cause of preventable visual impairment worldwide, affecting both adults and children. In the UK, incidence ranges from 3–4 per 10,000, consistent with global data showing anterior uveitis as the most prevalent subtype of intraocular inflammation (Buxton *et al*., 2023). Diagnosing anterior uveitis in children and young people (CYP) is challenging, as the disease is often asymptomatic; delayed detection may lead to irreversible ocular complications such as band keratopathy, glaucoma, cataracts, and macular and optic disc oedema (Maghsoudlou *et al*., 2025; Maleki *et al*., 2022; Standardization of Uveitis Nomenclature Working Group, 2005).

Anterior uveitis is typically diagnosed by identifying and quantifying inflammatory cells present in the anterior chamber via slit-lamp biomicroscopy (Maleki *et al*., 2022; Standardization of Uveitis Nomenclature Working Group, 2005). The Standardisation of Uveitis Nomenclature (SUN) Working Group defines inflammation severity based on the number of cells observed in a 1 × 1 mm bright beam, defining six cell-density categories (Duplechain *et al*., 2025; Standardization of Uveitis Nomenclature Working Group, 2005).

- Grade 0: <1 cell
- Grade 0.5+(Trace): 1–5 cells
- Grade 1+: 6–15 cells
- Grade 2+: 16–25 cells
- Grade 3+: 26–50 cells
- Grade 4+: >50 cells

Since its invention in 1911, the slit lamp has evolved through multiple illumination systems, from Nernst Glower bulbs to tungsten and halogen bulbs (Timoney and Breathnach, 2013; Zeiss Campus, n.d.). Light is generated by heating a tungsten filament coated with metal oxides and focused through a slit beam to produce a sharply defined rectangular beam. While providing sufficient illumination, the substantial heat generated by the bulb gradually vaporises the tungsten filament into black sooty deposits that condense in the bulb’s interior (Zeiss Campus, n.d.). This gradually reduces light output and filament mass, leading to inconsistent illumination and a shorter light span.

The first commercially available light-emitting diode (LED) slit-lamp was introduced in 2010, marking a paradigm shift in ophthalmic instrumentation. LED technology offers improved brightness consistency, reduced heat generation, and a significantly extended life span, often exceeding 30,000 hours while typically not requiring routine replacement during the device’s service life (Kolomeyer *et al*., 2010). Despite LED slit lamps becoming prevalent, tungsten units remain in use, raising the potential issue of variability in light quality between different bulbs.

Slit-lamp biomicroscopy is routinely performed across multidisciplinary eye services, including orthoptists, optometrists and ophthalmologists. In National Health Service (NHS) clinics, clinicians often have limited control over the available slit-lamp models, and mixed use of LED and tungsten systems may introduce inter-device variability due to differences in illumination quality. The clinical observation of many experienced practitioners found that some slit-lamps, even of the same model, enhanced the visibility of inflammatory cells better than others, hence motivating this project to investigate potential diagnostic variability.

While previous studies have explored the general advantages of LED over traditional illumination systems and compared a limited number of slit lamps (Wong, Nugent and Vargas-Martín, 2009), this study aims to assess all slit lamps within a tertiary eye care centre to systematically quantify device-to-device variability and determine whether such variability impacts diagnostic accuracy, particularly in detecting anterior uveitis.

## Materials and Methods

### Slit lamps

43 Haag-Streit BQ900 slit lamps (21 tungsten-based and 22 LED) located in the ophthalmology outpatient department at a tertiary ophthalmology centre in the UK were evaluated. Tungsten-based slit lamps in this study were equipped with low-voltage tungsten-halogen bulbs (model: HS 900-930, 6 V, 4.5 A, P44s base; Dr. Fischer, Haag-Streit). Lux emissions were quantitatively measured using a calibrated Dual-Range Light Meter (Traceable®, Webster, TX, USA) under three standardised conditions (open aperture, 1 × 1 mm, and 45°) with/without room lighting: All measurements were conducted by a single operator to minimise variability. Reference numbers and latest service date were recorded from the engineer’s service logbook to identify potential correlations between maintenance history and light output variability. Lux measurements were recorded at the optimal focal point under 16× magnification (Figure 1a).

**Figure 1:**
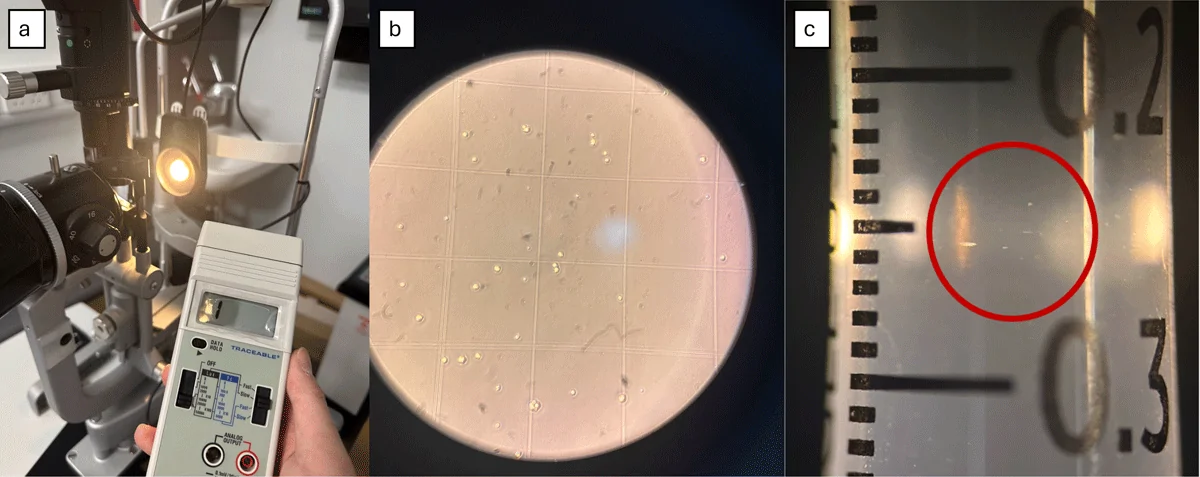
Experimental setup and imaging used in slit-lamp performance evaluation. **(a)** Lux measurements recorded using a calibrated photometer. **(b)** Simulated inflammatory cells visualised in suspension under a haemocytometer grid. **(c)** Appearance of low-grade cells under a 1 × 1 mm slit beam at 16× magnification.

### Simulated anterior chamber

A simulated anterior chamber model was developed using cornstarch microparticles suspended in filtered ethanol. Cornstarch granules are intrinsically polydisperse, with typical diameters in the order of 10–20 µm (mean 15 µm) in diameter (Jane *et al*., 1992), overlapping the size scale of typical inflammatory cells observed in anterior uveitis, including neutrophils, lymphocytes, and macrophages (Duplechain *et al*., 2025). The particulate was selected as a pragmatic light scattering surrogate within a clinically relevant size range. In contrast to monodisperse polystyrene microspheres, which offer size uniformity, a polydisperse suspension provides size heterogeneity that more closely reflects the variability encountered during clinical slit-lamp grading, which has been associated with observer variability (Wong, Nugent and Vargas-Martín, 2009).

To simulate the anterior chamber depth for visual observations, 1 mL Luer Lock (SOL-M™) was used, representing the smallest syringe readily available. Preliminary tests revealed that larger syringes distorted observations by introducing visual artefacts and increasing apparent particle density. The selected 1 mL syringes, with their thinner barrel walls and narrowest internal diameter (0.7 cm), minimised these optical illusions and thereby provided a consistent viewing chamber. Previous pilot trials on 3-dimensional printed and glass eye models were discontinued due to excessive visible impurities noted when viewed under high magnification.

Six solutions were prepared according to the SUN criteria, targeting mid-range cell concentrations for each grading category: approximately 80 cells/mm^3^ for grade 4+, 40 cells/mm^3^ for grade 3+, 20 cells/mm^3^ for grade 2+, 10 cells/mm^3^ for grade 1+, and 5 cells/mm^3^ for grade 0.5+. SUN grade 0 was filled with only ethanol after passing through a 5-micron filter, serving as the standardised control without any particles added. A stock suspension was serially diluted to produce target concentrations, calculated by basing the known anatomical depth (approximately 3 mm) of the anterior chamber and scaled proportionally to the volume of the syringes used (Feng *et al*., 2011). The particle counts were independently verified using a haemocytometer by two investigators to confirm particle concentrations aligned within the intended SUN grading parameters (Figure 1b).

Unlike studies employing automated tracking systems or optical coherence tomography (OCT) for quantifying inflammatory cells, we adopted direct visual observation to reflect inherent real-world diagnostic variability (Wong, Nugent and Vargas-Martín, 2009). Cell counting and grading were performed by 13 masked observers with varying ophthalmological clinical experience, reflecting real-world diagnostic variability and generalisability of findings.

The simulated chambers were graded from 0 to 4+ using a 1 × 1 mm slit beam at maximum brightness and 16× magnification. Each test module was shaken vigorously for five seconds immediately before evaluation to ensure consistency and induce particle movement, mimicking the physiological dynamics of inflammatory cells in the aqueous humour. A laboratory prong held the simulated chambers securely in position to maintain examination consistency. Observers assessed each simulated chamber using SUN criteria, counting and grading cells accordingly (Figure 1c). The two slit lamps used for the simulated chamber comparison were selected from the lower end of measured illuminance (one tungsten, one LED) to evaluate whether clinically encountered low-output performance could influence low-grade inflammation detection. Grading was performed with room lighting off at each of the two testing locations. Ambient light under these conditions did not exceed 5 lux, a negligible contribution relative to the slit-lamp brightness levels being compared.

To ensure observers and recorders were masked, all simulated chambers were coded with a geometric shape, with findings recorded separately. The primary aim was to assess the relative diagnostic performance of different slit lamps under standardised conditions and not just an absolute quantification of anterior chamber cells. Observed cell counts served as a comparative metric to evaluate inter-device variability. Data analysis included coefficient of variation (CV) and paired t-tests comparing inter-device consistency and diagnostic sensitivity.

Statistical analyses were performed in Microsoft Excel (Microsoft 365; Version 2602, Microsoft Corporation, Redmond, WA, USA), and figures were generated in GraphPad Prism (Version 8.0.2, GraphPad software, San Diego, CA, USA).

### Patient and public involvement

Patients or the public were not involved in the design, conduct, reporting or dissemination of this study. Observers were clinicians and trainees assessing simulated anterior chambers, and no patient-facing procedures or identifiable data were collected.

## Results

### Slit-lamp illumination variability

To establish a photometric baseline, maximum brightness output was measured for each slit lamp. While this does not reflect standard clinical practice, it serves as a useful benchmark to assess the theoretical output capacity of each device. The results illustrate the variability among all slit lamps used in clinical settings.

Under full brightness (open aperture) at a front-facing angle, 14 of the 22 (63.6%) LED slit lamps exceeded the upper detection limit of the photometer (>200,000 lux). This produced a ceiling effect (right-censoring), which limited statistical comparisons under this setting. Under identical settings, all tungsten slit lamps measured within the measurable range (23,500–126,400 lux), while LED lamp emissions spanned from 97,400 to values exceeding 200,000 lux. LED devices more frequently exceeded the instrument ceiling, indicating a higher maximum output capacity. These settings were not included in direct comparisons due to the data truncation. Subsequent statistical comparisons were limited to two alternative settings: full aperture at a 45° angle, which represented peak brightness under standardised orientation, and 1 × 1 mm aperture at 45°, which reflected SUN-recommended diagnostic conditions. Both settings fell within the quantifiable range of the photometric instrument and allowed for valid intergroup comparison (Table 1).

**Table 1:** Summary of lux recordings for full brightness and 1 × 1 mm, both at 45°, stratified by bulb type.


APERTURE/FILTER	ROOM LIGHTS	BULB TYPE	MEAN LUX	SD	MEDIAN (IQR)	RANGE (MIN–MAX LUX)	COEFFICIENT OF VARIATION (CV)

Full brightness (Open Aperture) 45°	ON	LED	142855	35689	150700 (62300)	78100–197000	24.98%

	ON	Tungsten	55143	23970	50500 (36000)	21500–116900	43.47%

	OFF	LED	141509	35029	150600 (62175)	77800–196900	24.75%

	OFF	Tungsten	53805	23871	49300 (28900)	21300–116000	44.37%

Full brightness (1 × 1 mm) 45°	ON	LED	1413	525	1310 (568)	700–2570	37.22%

	ON	Tungsten	1011	480	1040 (630)	290–2240	47.41%

	OFF	LED	1275	503	1205 (435)	540–2440	39.44%

	OFF	Tungsten	793	438	720 (500)	240–2070	55.21%


Under full brightness and an open aperture at a 45° angle, all 43 slit lamps, regardless of bulb type, demonstrated considerable variability in lux output, indicating intrinsic variability even within the same model line (Haag-Streit BQ900).

Statistically significant differences in brightness were observed between LED and tungsten lamps under both ambient lighting conditions. With room lighting off, LED devices emitted a mean brightness of 141,509 lux (SD: 35,029), compared to 53,805 lux (SD: 23,871) from tungsten models (t(37) = 9.63, p < 0.0001). When measured with room lighting on, capturing background illumination, the disparity remained substantial: LED slit lamps produced a mean of 142,855 lux (SD: 35,689), versus 55,143 lux (SD: 24,056) from tungsten devices, again yielding a significant result (t(37) = 9.50, p < 0.0001). This large effect (Cohen’s d = 2.87) confirms the markedly greater brightness of LED slit lamps versus tungsten slit lamps with minimal overlap. Despite producing nearly three times the lux level of tungsten models, LED demonstrated lower relative variability, with a coefficient of variation (CV) of approximately 25% compared to 44% in the tungsten group. Under standardised diagnostic conditions for anterior uveitis (1 × 1 mm aperture at 45°), LED slit lamps emitted significantly higher lux levels than their tungsten counterparts, regardless of ambient room lighting (Figure 2a). With room light off, LED devices produced a mean brightness of 1275 lux (SD: 503) compared to 793 lux (SD: 438) for tungsten models, effectively 1.6× brighter (Figure 2b). This difference was statistically significant (independent two-sample t-test, t(41) = 3.35, p = 0.0017), confirming a significant advantage for LED illumination under typical diagnostic settings. When measured with room light on, the mean lux remained higher in LED devices (1413 lux, SD: 526) than in tungsten (1011 lux, SD: 480), again reaching statistical significance (t(41) = 2.62, p = 0.012). Relative variability remained lower in the LED group (CV: 39.4%) versus tungsten (CV: 55.2%).

**Figure 2:**
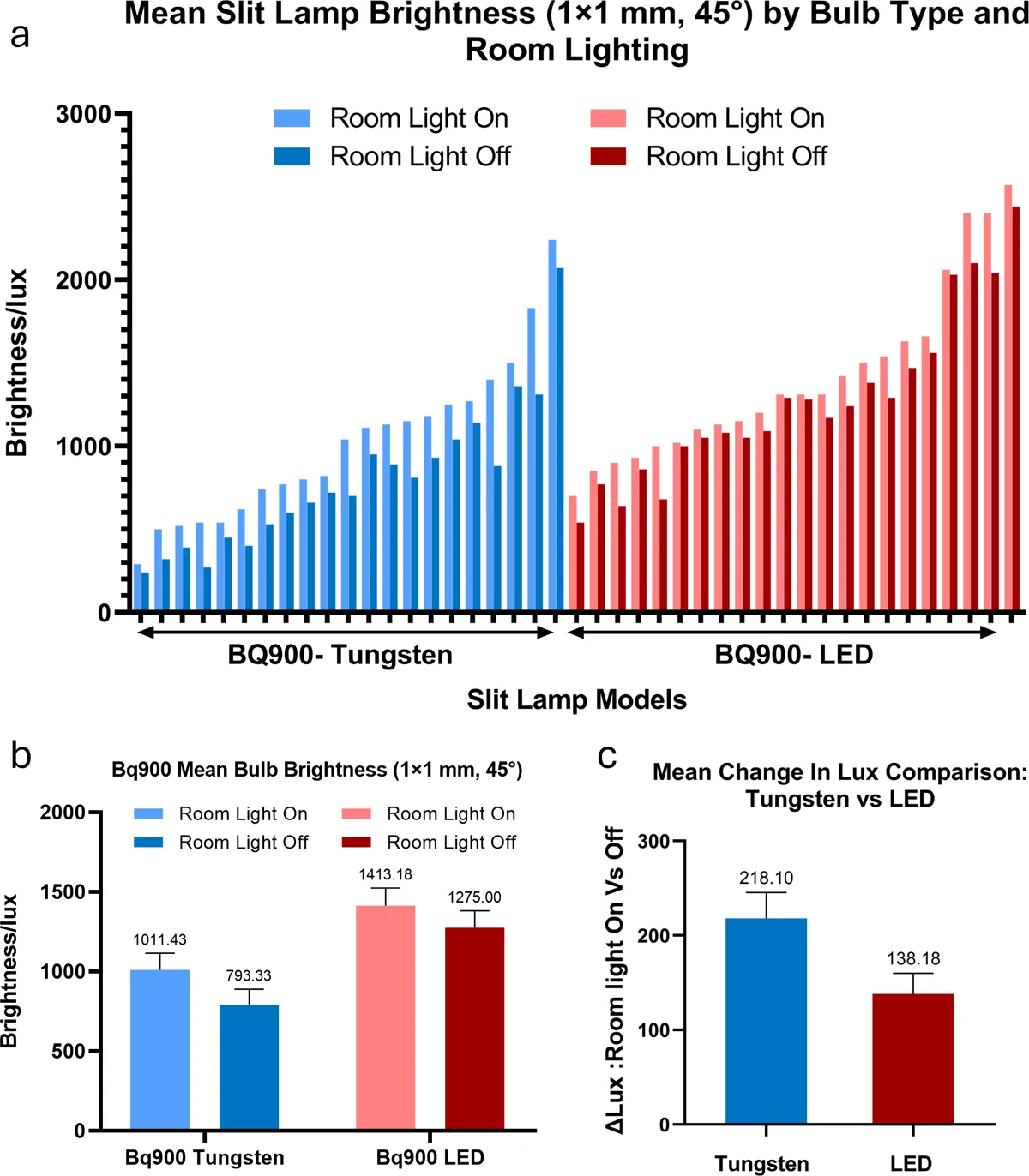
**(a)** Individual slit-lamp lux output under diagnostic settings. Lux measurements from BQ900 slit lamps (1 × 1 mm, 45° beam) with room lights ON and OFF, stratified by bulb type. LED lamps consistently emitted higher lux and showed reduced variability; ambient lighting had a greater impact on tungsten models. **(b)** Mean brightness of BQ900 slit lamps under room light ON and OFF. LED lamps produced higher mean lux output than tungsten models under both lighting conditions. Error bars indicate standard error of the mean (SEM). **(c)** Mean lux difference between room lighting conditions. The change in measured lux between room light ON and OFF for LED and tungsten lamps. LED models exhibited lower sensitivity to ambient lighting, indicating greater consistency. Data shown as mean ± SEM.

### Comparisons of the mean difference in lighting conditions

Lux recordings were measured with room lighting switched on and off, simulating typical outpatient environments. Both LED and tungsten lamps showed increased lux readings with the room lighting on. However, tungsten devices demonstrated a significantly greater increase, with a mean lux increase of 218.1 compared to 138.2 in LEDs (p = 0.027, Cohen’s d = 0.7) (Figure 2c).

### Service history and tungsten bulb replacement

Manufacturer service dates were reviewed in all 43 slit lamps, ranging from January 2025 to September 2025, with the majority completed between May and July 2025. Bulb replacement dates were available for all tungsten-based units (n = 21), enabling analysis of light output in relation to bulb age. Service logs were also available for LED slit lamps but only documented routine maintenance, as LED modules had not yet required bulb replacement within their expected lifespan.

Despite the broad service interval, no clear correlation was observed between bulb age and measured light output. Tungsten bulb replacement occurred between September 2019 and July 2024, corresponding to 51–1880 days prior to the lux measurement. The majority of replacements occurred in 2024 (59.1%), followed by 2023 (18.2%), 2022 (18.2%), and one in 2019 (4.5%). When replacement age (in days) was plotted against corresponding lux output, no significant correlation was found (Pearson *r* = –0.25, *p* = 0.25; Spearman *r_s_* = –0.35, *p* = 0.11). These findings suggest that duration since bulb replacement was not a reliable predictor of illumination performance, likely reflecting differences in device usage pattern, operating hours or undocumented periods of continuous-on that could not be retrospectively accounted for.

### Simulated anterior chamber testing

Given the observed variability in slit-lamp brightness, we investigated whether this translated into differences in diagnostic performance using the simulated anterior chamber model. Thirteen masked-observers assessed six cell grades using two clinically relevant slit-lamps: an LED model (680 lux) and a tungsten model (390 lux), both tested under standardised conditions (1 × 1 mm, 45°, room light–off). Despite the relatively narrow lux differential, LED illumination demonstrated significantly higher sensitivity for detecting lower SUN grade inflammation (Table 2).

**Table 2:** Comparison of LED vs tungsten slit-lamp detection of simulated inflammatory cells across SUN grades 0–4+ (1 × 1 mm, 45° room light off). Mean and median values reported due to non-normal paired differences. LED illumination showed significantly higher detection at SUN grades 0.5+ and 1+, with no significant difference at higher grades.


Grade	Mean Cell Count		Median Cell Count		Mean Diff	Median Diff	SD of paired diff	p-value	95% CI

	LED	TUNGSTEN	LED	TUNGSTEN					

0	0.15	0	0.00	0.00	0.15	0.00	0.38	0.16541	[–0.07, 0.38]

0.5	2.23	0.77	2.00	1.00	1.46	2.00	0.88	**0.00006**	**[0.93, 1.99]**

1	5.77	4.15	6.00	4.00	1.62	2.00	2.53	**0.04033**	**[0.08, 3.15]**

2	16.46	14.62	18.00	14.00	1.85	2.00	3.78	0.10209	[–0.43, 4.12]

3	23.31	19.54	22.00	17.00	3.77	4.00	9.18	0.16474	[–1.78, 9.32]

4	41.46	38.38	40.00	40.00	3.08	0.00	10.26	0.30077	[–3.12, 9.28]


At SUN grade 0, mean cell counts were low overall (LED: 0.15 cells vs tungsten: 0 cells; p = 0.165), with two of 13 observers reporting a single false-positive cell under LED illumination and no false-positive reports under tungsten illumination. At SUN grade 0.5+, mean cell counts were significantly higher with LED illumination compared to tungsten (2.23 vs 0.77 cells; *p* = 0.00006; mean difference 1.46 cells, 95% CI: 0.93–1.99). This advantage persisted at grade 1+, with LED yielding 5.77 cells versus 4.15 with tungsten (*p* = 0.0403; mean difference 1.62 cells, 95% CI: 0.08–3.15). Although LED slit lamps continued to yield higher mean cell counts at SUN grades 2+ to 4+, the differences were not statistically significant, reflecting that the inflammation at these higher grades is more conspicuous and readily detectable under both lighting conditions (Figure 3a).

**Figure 3:**
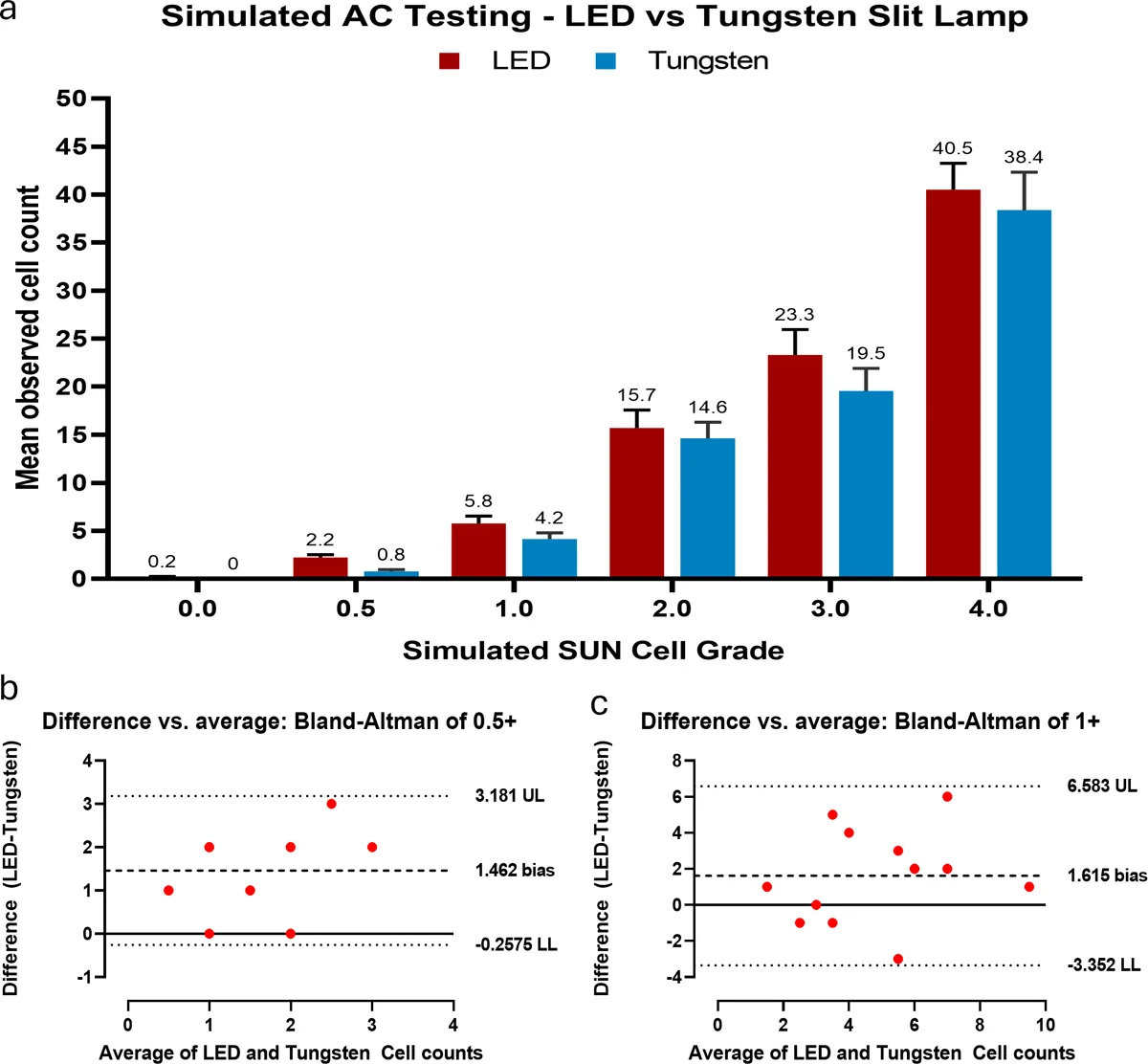
**(a)** Mean inflammatory cell counts by SUN grade under simulated testing. Observed cell counts using LED and tungsten slit lamps (1 × 1 mm, 45° beam, lights OFF). LED lamps detected significantly more cells at lower SUN grades (0.5+ and 1+). Data shown as mean ± SEM. **(b, c)** Bland–Altman analysis of LED vs. tungsten slit lamp detection. Plots show the difference in cell counts (LED–tungsten) against the mean cell count per observer at SUN grades 0.5+ and 1+. The mean bias reflects improved detection with LED illumination. 95% Limits of agreement are shown.

Bland–Altman plots were generated for all SUN grades to assess agreement between LED and tungsten slit-lamp measurements. However, only plots for grades 0.5+ and 1+ are presented (Figures 3b and 3c), where statistically significant differences were identified with paired *t*-tests in mean cell counts. At these lower inflammation levels, the plots revealed a consistent positive bias toward higher cell detection with LED illumination, with all differences falling within the 95% limits of agreement, suggesting a systematic, clinically meaningful diagnostic advantage.

Overall, these findings indicate that higher illuminance is associated with improved sensitivity in detecting low-grade subtle anterior chamber inflammation in this simulated model. At higher SUN grades, although LED illumination still showed a positive bias, the clinical relevance diminished due to overlapping limits of agreement and reduced dependency on optimal lighting for visualisation.

## Discussion

While LED and tungsten slit lamps differ fundamentally in illumination technology, this study found substantial variability in light output even among devices of the same model, with tungsten slit lamps exhibiting greater inconsistency. No significant correlation was observed between light output, bulb age, or scheduled service intervals. The discrepancies can be plausibly attributed to real-world variable clinical usage patterns; some machines were used more frequently and for longer hours than others, and intermittent periods of unintentional continuous illumination may accelerate filament degradation in tungsten slit lamps.

Despite the variability, in the simulated anterior chamber model, the LED slit lamp demonstrated higher detection at low SUN grades than the tungsten slit lamp, consistent with the higher and more stable illuminance observed across the LED cohort. This is clinically important, especially in paediatric or asymptomatic uveitis, where subtle inflammation may otherwise go undetected. The greater brightness uniformity, reduced thermal output, and extended operational lifespan of LED slit-lamp technology further support their preferential use for anterior segment assessment. The lower coefficient of variation across LED devices also indicates more consistent output between individual units, providing more predictable illumination regardless of which lamp a patient is examined under. Tungsten devices were additionally more affected by ambient lighting, showing a greater change in measured lux between room-light conditions; as many consultation rooms do not achieve complete darkness, this reinforces the greater reliability of LED lamps across lighting environments.

Colour temperature is another potential factor to consider; traditional tungsten bulbs emit a warmer light (~2700 Kelvin), whereas LEDs typically produce a cooler white light (~6500 K). Cooler colour temperature illumination is associated with improved contrast and fine-detail perception; however, there is currently no evidence of the influence of colour temperature on anterior chamber cell visibility, and evaluating spectral effects was beyond the scope of this study (Rossi *et al*., 2024; Soltic and Chalmers, 2019).

### Clinical implications

These findings emphasise the importance of standardised slit-lamp illumination in uveitis service. Significant variability between devices, even within the same model, highlights the need for routine illumination audits and the establishment of a minimum lux output threshold. Where feasible, retrofitting or upgrading older tungsten slit lamps to LED modules may enhance diagnostic sensitivity, consistency and inter-observer reproducibility.

This is particularly relevant in paediatric and high-risk settings where early detection is critical, such as in juvenile idiopathic arthritis-associated uveitis surveillance programmes. Even when early inflammation does not immediately alter treatment, its detection provides clinicians with crucial situational awareness, supports shorter follow-up intervals, closer monitoring of therapeutic adherence, and timely escalation when disease activity increases. Conversely, missed detection from insufficient illumination may delay diagnosis and cause preventable visual loss. Optimising slit-lamp performance and ensuring consistent illumination standards directly enhance early intervention, improve long-term outcomes and a more proactive, patient-centred approach to disease management.

### Limitations

The primary limitation is the use of a simulated anterior chamber model, which cannot replicate the full anatomical and optical complexity of the human eye. Physiological factors such as iris pigmentation and corneal transparency may all influence cell visibility in vivo. Ethanol and cornstarch differ from aqueous humour and biological cells in viscosity, physical structure, and light-scattering behaviour, which may influence particle behaviour relative to true inflammatory cells in vivo. Additionally, subtle differences in focal depth, beam alignment, observer position and viewing angle may affect cell detection. Nevertheless, slit-lamp performance was evaluated under identical, standardised conditions across all devices. Importantly, Bland–Altman analysis supported the validity of relative diagnostic differences in cell detection, particularly at lower SUN grades, where the observed disparities reflected clinically meaningful variability.

Another limitation was the small observer number (n = 13), reflecting practical constraints during routine clinical hours. While observer technique variability is inherent in real-world clinical examinations, the within-subject comparison design, where each participant evaluated both slit-lamp types under identical conditions, helped to minimise inter- and intra-observer variability, thereby strengthening the reliability and allowing relative comparisons.

At SUN Grade 0, two of 13 observers reported a single false-positive cell under LED illumination, with none under tungsten; this isolated finding may partly reflect the forced-choice structure of the simulated testing set, in which observers were aware that a Grade 0 chamber was included, an expectation not present in routine clinical examination. Inter-observer variation, an inherent and unavoidable feature of subjective slit-lamp assessment, may also have contributed.

Additionally, only the dimmest tungsten and LED slit lamps were used for the simulated anterior chamber comparison, selected to evaluate diagnostic performance under the lowest clinically encountered illuminance. While this approach targeted the setting in which illumination-related differences were most likely to be clinically meaningful, mid-range and high-output devices were not directly compared with the simulated anterior chamber model.

Finally, the absolute lux values reported may not fully represent the maximum light output of some of the LED slit lamps, limited by the photometer’s sensor size, angular sensitivity, and detection threshold. The ceiling effect observed during full brightness testing among some LED devices further constrained quantification. However, since all recordings were conducted under uniform and reproducible testing conditions (fixed distance, angle, and aperture), the proportional differences observed across devices remained robust indicators and reflect meaningful true performance disparities.

## Conclusion

This study demonstrates that slit lamps are not functionally equivalent. Significant variability in light emission, particularly between LED and tungsten models, potentially affects the reliability of anterior segment examination. In a controlled simulated environment, the LED slit lamp assessed delivered consistently higher and more stable illumination, which translated into improved sensitivity in the detection of low-grade anterior chamber inflammation. These findings emphasise equipment standardisation and support LED illumination to improve diagnostic accuracy, consistency and timely intervention.

## References

[1] Buxton, A., Morgan, C., Thomason, R. and Currie, C. (2023) ‘EPH91 The incidence of uveitis in the United Kingdom 2004–2021’, Value in Health, 26(Suppl 12), p. S219. Available at: 10.1016/j.jval.2023.09.1133

[2] Duplechain, A., Conrady, C.D., Patel, B.C. and Baker, S. (2025) ‘Uveitis’, in StatPearls [Internet]. Treasure Island, FL: StatPearls Publishing. Available at: https://www.ncbi.nlm.nih.gov/books/NBK540993/ (Accessed: 12 May 2025).

[3] Feng, M.T. *et al*. (2011) ‘Anterior chamber depth in normal subjects by rotating Scheimpflug imaging’, Saudi Journal of Ophthalmology, 25(3), pp. 255–259. Available at: 10.1016/j.sjopt.2011.04.005 PMC3729361 23960933

[4] Jane, J., Shen, L., Wang, L. and Maningat, C.C. (1992) ‘Preparation and properties of small-particle corn starch’, Cereal Chemistry, 69(3), pp. 280–283.

[5] Kolomeyer, A.M. *et al*. (2010) ‘The use of LED illumination in high-definition tele-stereo slit-lamp biomicroscopy’, Investigative Ophthalmology & Visual Science, 51, ARVO E-Abstract 1022.

[6] Maghsoudlou, P., Epps, S.J., Guly, C.M. and Dick, A.D. (2025) ‘Uveitis in adults: a review’, JAMA, 334(5), pp. 419–434. Available at: 10.1001/jama.2025.4358 40434762

[7] Maleki, A. *et al*. (2022) ‘Pediatric uveitis: a comprehensive review’, Survey of Ophthalmology, 67(2), pp. 510–529. Available at: 10.1016/j.survophthal.2021.06.006 34181974

[8] Rossi, S. *et al*. (2024) ‘Influence of lighting on visual performance’, Arquivos Brasileiros de Oftalmologia, 87(3), e20230257. Available at: 10.5935/0004-2749.2023-0257 PMC11627233 38716966

[9] Soltic, S. and Chalmers, A. (2019) ‘Optimization of LED lighting for clinical settings’, Journal of Healthcare Engineering, 2019, Article ID 5016013. Available at: 10.1155/2019/5016013 PMC6732584 31534645

[10] Standardization of Uveitis Nomenclature Working Group (2005) ‘Standardization of uveitis nomenclature for reporting clinical data: Results of the First International Workshop’, American Journal of Ophthalmology, 140(3), pp. 509–516. Available at: 10.1016/j.ajo.2005.03.057 PMC8935739 16196117

[11] Timoney, P.J. and Breathnach, C.S. (2013) ‘Allvar Gullstrand and the slit-lamp 1911’, Irish Journal of Medical Science, 182, pp. 301–305. Available at: 10.1007/s11845-012-0873-y 23264115

[12] Wong, I.G., Nugent, A.K. and Vargas-Martín, F. (2009) ‘The effect of biomicroscope illumination system on grading anterior chamber inflammation’, American Journal of Ophthalmology, 148, pp. 516–520.e2. Available at: 10.1016/j.ajo.2009.04.027 19541282

[13] Zeiss Campus (n.d.) ‘Tungsten-halogen light sources’ [Internet]. Available at: https://zeiss-campus.magnet.fsu.edu/print/lightsources/tungstenhalogen-print.html (Accessed: 10 May 2025).

